# The Effect of Multiple Evolutionary Selections on Synonymous Codon Usage of Genes in the *Mycoplasma bovis* Genome

**DOI:** 10.1371/journal.pone.0108949

**Published:** 2014-10-28

**Authors:** Jian-hua Zhou, Yao-zhong Ding, Ying He, Yue-feng Chu, Ping Zhao, Li-ya Ma, Xin-jun Wang, Xue-rui Li, Yong-sheng Liu

**Affiliations:** State Key Laboratory of Veterinary Etiological Biology, National Foot-and-Mouth Disease Reference Laboratory, Lanzhou Veterinary Research Institute, Chinese Academy of Agricultural Sciences, Lanzhou, Gansu, P.R. China; Beijing Institute of Genomics, Chinese Academy of Sciences, China

## Abstract

*Mycoplasma bovis* is a major pathogen causing arthritis, respiratory disease and mastitis in cattle. A better understanding of its genetic features and evolution might represent evidences of surviving host environments. In this study, multiple factors influencing synonymous codon usage patterns in *M. bovis* (three strains’ genomes) were analyzed. The overall nucleotide content of genes in the *M. bovis* genome is AT-rich. Although the G and C contents at the third codon position of genes in the leading strand differ from those in the lagging strand (p<0.05), the 59 synonymous codon usage patterns of genes in the leading strand are highly similar to those in the lagging strand. The over-represented codons and the under-represented codons were identified. A comparison of the synonymous codon usage pattern of *M. bovis* and cattle (susceptible host) indicated the independent formation of synonymous codon usage of *M. bovis*. Principal component analysis revealed that (i) strand-specific mutational bias fails to affect the synonymous codon usage pattern in the leading and lagging strands, (ii) mutation pressure from nucleotide content plays a role in shaping the overall codon usage, and (iii) the major trend of synonymous codon usage has a significant correlation with the gene expression level that is estimated by the codon adaptation index. The plot of the effective number of codons against the G+C content at the third codon position also reveals that mutation pressure undoubtedly contributes to the synonymous codon usage pattern of *M. bovis*. Additionally, the formation of the overall codon usage is determined by certain evolutionary selections for gene function classification (30S protein, 50S protein, transposase, membrane protein, and lipoprotein) and translation elongation region of genes in *M. bovis*. The information could be helpful in further investigations of evolutionary mechanisms of the *Mycoplasma* family and heterologous expression of its functionally important proteins.

## Introduction

*Mycoplasma* is regarded as the smallest self-replicating microorganism without a cell wall, and it is classified in the family Mycoplasmataceae [1]. *Mycoplasma* is unusual among bacteria in that most species require sterols for the stability of their cytoplasmic membrane. The lack of a cell wall leads to several specific properties of mycoplasmas such as sensitivity to osmotic shock and detergents and the formation of odd fried-egg-shaped colonies. Mycoplasmas are essentially composed of three organelles, the cell membrane, ribosomes, and a circular double-stranded DNA molecule that is tightly packed. This life form (including more than 100 identified species) widely exists in nature by means of a saprotrophic or parasitic pathway [2]. Among these *Mycoplasma* species, *Mycoplasma bovis* is pathogenic in cattle and serves as a major agent resulting in respiratory disorders, arthritis, and mastitis [3]. This etiological agent can play an obvious role in impairing the dairy industry because many antibiotics that target cell wall synthesis often fail to affect the self-replicating process of *Mycoplasma* [3]. Mycoplasmas generally possess a relatively small genome of about 10^6^ bp with coding sequences with low G+C content (<33%), which results in drastically reduced biosynthetic capabilities and explains their dependence on a host [4]. Although *M. bovis* is a simpler and smaller life form than other bacteria, the mechanisms of *M. bovis* pathogenicity remain largely unknown [5]. With the development of sequencing technology for microorganisms, the three complete genomes of *M. bovis* have been published, and the genomic annotation has identified some putative virulent genes [6], [7], [8], which are yet to be confirmed. These sequencing studies of the *M. bovis* genome assist researchers in scouring some useful genes that express virulence factors, membrane surface proteins, lipoproteins, and protease-related metabolic pathways.

With the largely available resources of the complete genome of *M. bovis*, taking advantage of the genetic information about this microorganism can assist in investigating the evolution and pathogenicity mechanisms of this organism. Except for analyzing the nucleotide and amino acid sequences, the synonymous codon use pattern can add additional genetic information about the organism and the reaction between the etiological agent and susceptible hosts [9], [10], [11], [12]. There are 61 canonical codons for 20 amino acids in nature, excluding three stop codons. Synonymous codons are not employed in equal and random frequencies, and the synonymous codon usage patterns often represent additional and essential genetic characteristics of most life forms, therefore, the ability to analyze synonymous codon usage bias for coding sequences plays an important role in revealing the underlying mechanisms behind synonymous codon usage and investigating gene evolution and function [13], [14]. Analyses of some microorganisms such as *Escherichia coli*, yeast, *Saccharomyces cerevisiae*, Chlamydiae, and spirochaetes revealed genetic information about the organisms that represents a mixture of different evolutionary factors such as mutation pressure from nucleotide composition, translation selection, intra-genomic variation, and strand-specific mutational bias [15], [16], [17], [18], [19]. The interaction of these factors may vary among different species depending on their evolutionary process. Here, based on the complete genome information about *M. bovis*, we carried out comprehensive analyses of its synonymous codon usage and nucleotide content to recognize roles of different factors in the evolutionary process. In addition, studies of synonymous codon usage patterns could better reveal *M. bovis* dynamics relevant to disease-control measures and explore reasons for its host and environmental adaptations.

## Materials and Methods

### Basic information about the *M. bovis* genome

Three complete genomes of strains (strain PG45 isolated from cow mastitis milk, strain HB0801 isolated from a lesioned bovine lung, strain Hubei-1 isolated from lung tissue of calf pneumonia) (accession no. NC_014760, NC_018077, and NC_015725) of *M. bovis* were downloaded from the NCBI Entrez Genomes Division site (http://www.ncbi.nlm.nih.gov) and were analyzed by means of synonymous codon usage and nucleotide composition to identify factors taking part in the evolutionary process of *M. bovis*. As for the background of the three strains, the genome of strain PG45 has a 29.3% GC content and contains 868 genes, the genome of strain HB0801 has 29.3% GC content and contains 873 genes, the genome of strain Hubei-1 has 29.3% GC content and contains 839 genes [6], [7], [8]. Among them, there were 57 genes for 30S ribosomal proteins, 89 genes for 50S ribosomal proteins, 68 genes for transposase (ISMbov 1–7), 100 genes for membrane proteins, and 128 genes for lipoproteins. These genes were applied to represent the genetic features of genes with a certain biological function in determining the synonymous codon usage pattern. According to the record of gene annotation in the three genomes of *M. bovis* in GenBank, the location information of each gene in the leading or lagging strand was processed and provided an available resource for estimating the role of the strand-specific mutational bias in the formation of synonymous codon usage for *M. bovis*. In addition, to estimate the similarity or deviation between *M. bovis* and cattle, the codon usage frequencies of cattle were obtained from the codon use database [20].

### Nucleotide composition statistics for genes in *M. bovis*

In many prokaryotic genomes and some archaeal genomes, asymmetry exists between the nucleotide compositions of the leading and lagging strands [21], [22]. Specifically, thymine (T) and guanine (G) are selected in the leading strand in high frequencies, whereas adenine (A) and cytosine (C) are highly selected in the lagging strand. This phenomenon is termed GC skew or AT skew [23]. Herein, depending on the ratio (GC skew) of (G − C)/(G+C) and the ratio (AT skew) of (A − T)/(A+T) [22], [24], a series of GC skew and AT skew data were calculated for codon positions 1, 2 and 3 to further investigate the effects of nucleotide composition bias at different nucleotide positions on the overall codon usage trends of a gene population.

### Calculating the relative synonymous codon usage values

The relative synonymous codon usage (RSCU) values for the 2380 genes in the three *M. bovis* genomes were calculated by the formula in a previous report [25]. Two genetic codons (AUG for Met and UGG for Trp) and three canonical stop codons should be excluded from the calculation of RSCU values. To identify the use bias of the 59 synonymous codons, a standard that codons with RSCU values >1.6 are over-represented and codons with RSCU values <0.6 are under-represented was introduced in this study, following the previous reports [11], [26]. Because principal component analysis (PCA) is a multivariate statistical analysis in which the values in the dataset are not independent [27], this method was introduced in this study to estimate the variation of synonymous codon usage of genes in the *M. bovis* genome and to analyze the similarity or deviation of the synonymous codon usage pattern of genes in the leading and lagging strand.

### Estimating effects of the overall codon usage of cattle on that of *M. bovis*

Based on codon usage frequencies of the genomes of cattle, the RSCU values for the organism were also calculated for the 59 synonymous codons by the formula for RSCU value.

To estimate the effect of the overall codon usage of cattle on *M. bovis*, a formula of *D(A,B)* was established to evaluate the potential role of the overall codon usage pattern of cattle in the formation of codon usage of *M.bovis*.
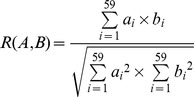





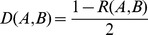
where *R(A,B)* is defined as a cosine value of an included angle between *A* and *B* special vectors representing the degree of similarity between *M. bovis* and cattle at the aspect of the overall codon usage pattern, *a_i_* is defined as the RSCU value for a specific codon in 59 synonymous codons of *M. bovis*, *b_i_* is termed as the RSCU value for the same codon of cattle. *D(A,B)* represents the potential effect of the overall codon usage of cattle on that of *M. bovis*, and *D(A,B*∈(0,1). The higher *D(A,B)* is, the stronger the effect of environment related synonymous codon usage patterns of cattle on that of M. bovis is.

### Other index concerning codon usage

To estimate the effect of GC content in the third position of a codon on synonymous codon usage, the G+C content at the third codon position (GC3s%) in each gene was calculated. GC3s% is regarded as a useful link with other codon usage indexes, such as the effective number of codons (ENC) and codon adaptation index (CAI), to represent the genetic features of synonymous codon usage. The ENC, which is a useful estimator of absolute codon bias, is a measure that identifies the overall codon use bias for a certain gene. ENC values have a range from 20 (when only one synonymous codon is chosen by the corresponding amino acid) to 61 (when all synonymous codons are used equally), and the lower the ENC value for one gene is, the stronger the overall codon usage bias for this gene [28]. CAI is also a useful tool for estimating codon usage bias. In microorganisms, lowly expressed coding sequences in the genome have a relatively low codon usage bias; in contrast, highly expressed coding sequences have a high codon usage bias [29].

### The synonymous codon usage in the translation elongation region of genes

We defined the translation elongation region of genes of *M. bovis* as the coding sequence, which is composed of 30 codon positions, with a range from the translation start codon to the 30^th^ codon position. The first 30 codons in all each genes obtained via manual edition were named as cod_1, cod_2, cod_3, …, cod_48, cod_49, and cod_50. Codons with the same name were mixed and recorded in FASTA format. Then, the new rearranged sequences were structured and calculated by the formula for the ENC value to represent the codon distribution at different positions.

### Statistical analysis

One-way analysis of variance (ANOVA) was used to estimate the differentiation of the nucleotide content of genes in the leading and lagging strands in the *M. bovis* genome. Correlation analysis involved in this study employed Spearman’s rank correlation (with the level of significance of p<0.05 or p<0.01) and was performed by SPSS version 11.5 for Windows.

## Results

### The nucleotide content of genes in the *M. bovis* genome

Overall, the nucleotide content of genes in the *M. bovis* genome is AT rich and GC poor. This feature is similar to that of other mycoplamsas [3], [4]. In detail, the T nucleotide content has a range from 47.41% to 13.06% (mean = 31.09%; SD = 3.59%), the A nucleotide content fluctuates from 58.62% to 25.53% (mean = 39.16%; SD = 11.24%), the C nucleotide content has a range from 27.42% to 6.25% (mean = 13.29%; SD = 9.25%), and the G nucleotide content has a range from 26.05% to 6.54% (mean = 16.46%; SD = 10.97%). One-way ANOVA for each nucleotide content of genes in the *M. bovis* genome revealed that the G nucleotide content is significantly different (p = 0.005) between the leading strand and the lagging strand, while the other three nucleotide contents have no significant difference. Furthermore, using the same assay for each nucleotide content in the first, second and third nucleotide position of codons, the C nucleotide content at the third codon position (C3s%) and the G nucleotide content at the third codon position (G3s%) are significantly different (p = 0.002 and 0.001, respectively) between the leading and the lagging strands. These results suggest that although the GC content of genes in the *M. bovis* genome is considerably low in the leading and lagging strands, the mutation pressure from GC content variation takes part in the formation of the nucleotide content of the *M. bovis* genome to a relatively small degree.

### The synonymous codon usage pattern of the *M. bovis* genome

The synonymous codon usage pattern of genes of the three strains of *M. bovis* does not represent the differentiation of the synonymous codon usage pattern (Table S1), suggesting that synonymous codon usage patterns of gene population of the three strains do not represent geography and genotypes. Furthermore, the first major variations (*f’_1_*>70%) of 59 synonymous codons of genes between the leading and lagging strands show that the synonymous codon usage pattern of the leading strand is similar to that of the lagging strand (Fig. S1). The under-represented codons include TT**C** for Phe; CT**C** and CT**G** for Leu; AT**C** for Ile; GT**C** and GT**G** for Val; TC**C** and TC**G** for Ser; CC**C** and CC**G** for Pro; AC**C** and AC**G** for Thr; GC**C** and GC**G** for Ala; TA**C** for Tyr; CA**G** for Gln; AA**C** for Asn; AA**G** for Lys; GA**C** for Asp; GA**G** for Glu; CG**C**, CG**A,** and CG**G** for Arg; and GG**G** for Gly. The over-represented codons include TT**T** for Phe, TT**A** for Leu, AT**T** for Ile, GT**T** for Val, TC**A** for Ser, CC**T** and CC**A** for Pro, AC**T** and AC**A** for Thr, GC**T** for Ala, CA**A** for Gln, GA**A** for Glu, and AG**A** for Arg (Table S1). The over-represented codons all end in T/A, while the under-represented codons all end in C/G, suggesting that nucleotide composition plays an important role in shaping the synonymous codon usage pattern for *M. bovis*. Comparisons of the synonymous codon usage patterns between *M. bovis* and cattle (susceptible host) revealed that cattle has a strong tendency to select many codons with G/C-ends, which are lost in *M. bovis* (CTG for Leu, CAG for Gln, CGG and AGG for Arg), and there are some synonymous codons with under- or over-represented polarization in the two organisms, such as TTA for Leu, GTG for Val, GCA and GCC for Ala, and CAA for Gln (Table S1). According to *D(A, B)* values reflecting the effect of overall codon usage pattern of cattle on that of *M. bovis*, the average of *D(A, B)* values is 0.224±0.001. The results, to some degree, reflect that although *M. bovis* has a strong tendency to infect and parasite cattle (specificity and restriction of host), *M. bovis*, which is a simple self-replication form, has a strong independence in its evolutionary processes at the aspect of synonymous codon usage.

### The overall codon usage pattern for *M. bovis*

**Figure 1:**
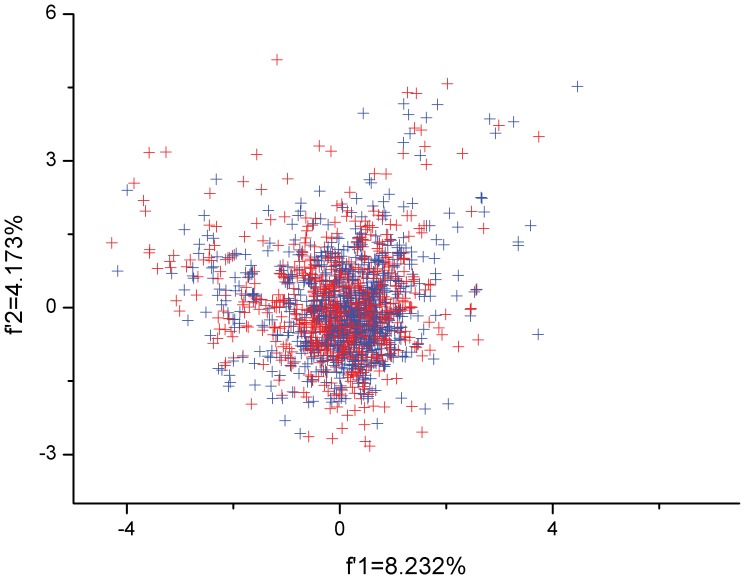
Plot of the first and second major axes generated by principal component analysis (PCA). The blue plots represent the genes in the leading strand of *Mycoplasma bovis*; the red plots represent the genes in the lagging strand of *M. bovis*.

The overall codon usage pattern of each gene in the *M. bovis* genome was determined by PCA based on RSCU values. The plots for codon usage patterns of genes in *M. bovis* were drawn by the first major variation (*f’_1_* = 8.232%) and the second major variation (*f’_2_* = 4.173%) of PCA on the plane. As for the role of strand-specific mutational bias in shaping the synonymous codon usage between the leading and the lagging strands, the large-scale overlay plots of both the leading strand and the lagging strand reflect that there was no factor influencing strand-specific mutational bias (Fig. 1). As for the effect of GC3s% on synonymous codon usage of *M. bovis*, the different grades of GC3s% divided the usage into different groups (Fig. S2), furthermore suggesting that the GC3s% plays a limited role in influencing synonymous codon usage.

To better understand the roles of base composition bias at different codon positions in the formation of synonymous codon usage, a series of correlations between composition bias and the major variation of codon usage was performed. The correlations between GC and AT skews and the two major variations obviously indicate that GC and AT composition bias slightly influence the synonymous codon usage of *M. bovis* (Table 1). Based on correlations between GC and AT skews at different codon positions and the two major variations, the effect of protein consideration with respect to the GC and AT skews at the first and second codon positions is weaker than that of mutation pressure caused by GC and AT skews at the third codon position on the formation of synonymous codon usage. These results imply that although the genome of *M. bovis* is generally AT-rich and GC-poor, the role of GC composition bias at the third codon position should not be neglected in the formation of synonymous codon usage of *M. bovis*.

**Table 1:** Nucleotide composition statistics for a gene population in *M.bovis.*

	*f’_1_*	*f’_2_*
GC Skew	r = −0.001 p>0.05	r = −0.047 p>0.05
AT Skew	r = −0.024 p>0.05	r = 0.011 p>0.05
GC1 Skew	r = −0.013 p>0.05	r = −0.013 p>0.05
AT1 Skew	r = 0.014 p>0.05	r = 0.014 p>0.05
GC2 Skew	r = −0.003 p>0.05	r = 0.003 p>0.05
AT2 Skew	r = −0.037 p>0.05	r = −0.037 p>0.05
GC3 Skew	r = 0.339*** p = 6.34×10^−65^	r = 0.005 p>0.05
AT3 Skew	r = −0.181*** p = 6.39×10^−19^	r = 0.081*** p = 7.21×10^−5^

*** means p<0.001.

**Figure 2:**
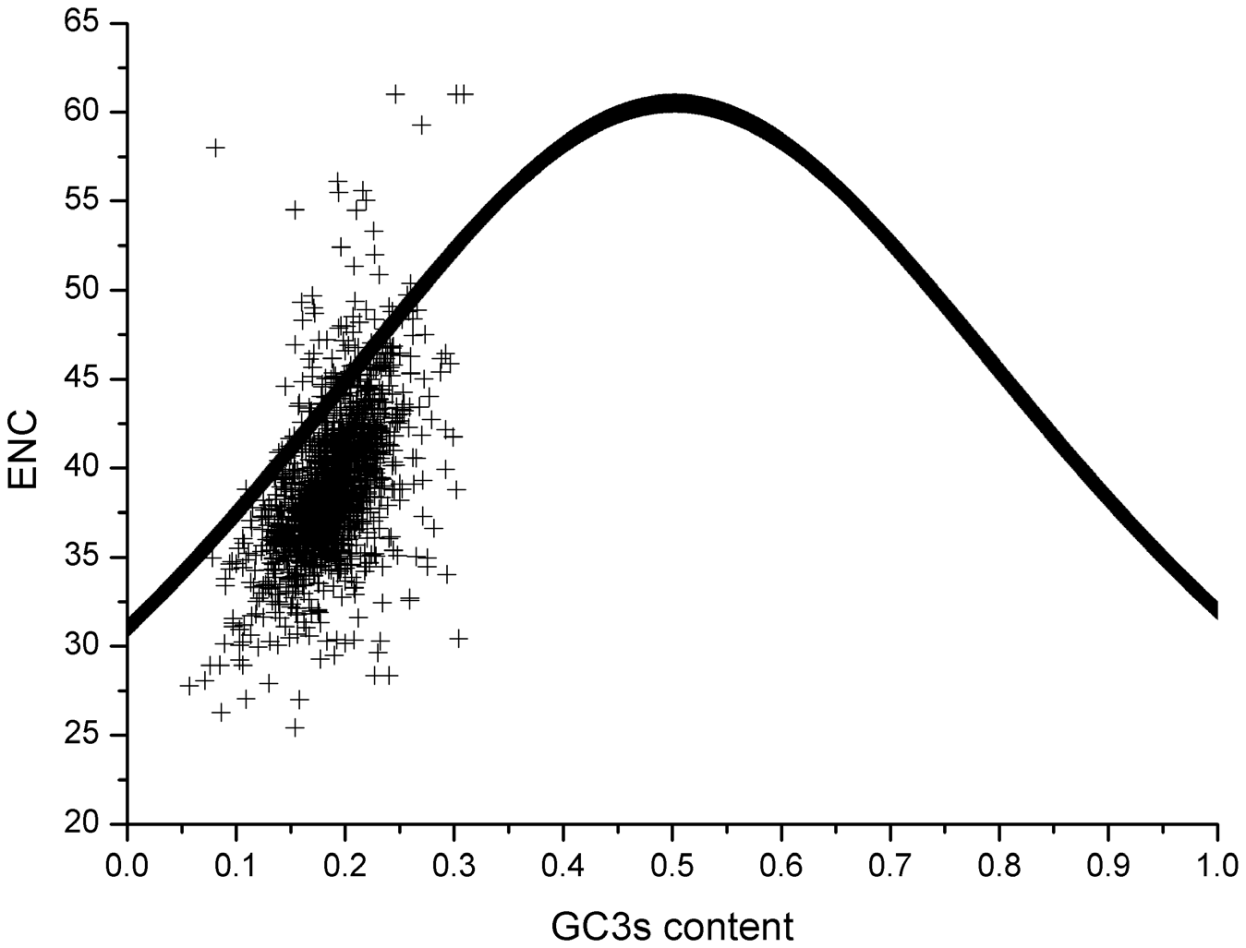
The effective number of codons (ENC) values for genes of *M. bovis*. The black plot represents the ENC value against the G+C content at the third codon position (GC3s%) of each gene of *M. bovis*. The continuous black curve indicates the expected curve between the ENC and GC3s% in random codon usage.

The plot of the GC3s% against the ENC value shows the overall codon usage pattern in the genome of a certain target organism. According to the standard of estimating the multiple factors that affect the overall codon usage [28], the phenomenon of scattered dots on the expected curve indicates that nucleotide compositional constraints are the single factor in the codon usage pattern under no other selection factors; in contrast, the phenomenon of scattered dots around the expected curve suggests that some other selection factors rather than compositional constraints influence the codon usage pattern. As for the plot of the ENC value vs. GC3s% for the *M. bovis* genome, most plots fail to follow the expected curve, and the fluctuation of GC3 content of genes in *M. bovis* has a limited effect on the overall codon usage pattern (Fig. 2). Furthermore, the correlation between the ENC value and the first major variation (*f’_1_*) is relatively low (r = 0.45, p<0.001) (Fig. S3). The results imply that both nucleotide composition and other evolution factors play roles in the overall codon usage in the *M. bovis* genome.

**Figure 3:**
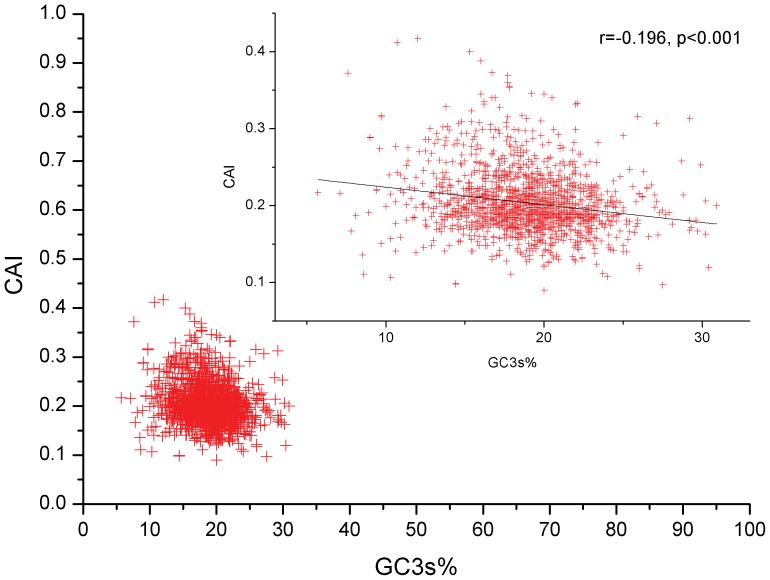
The codon adaptation index (CAI) values for genes of *M. bovis*. The correlation between CAI values and GC3s% was estimated by means of Spearman’s rank. The red plot represents the CAI value against the GC3s% for each gene, and the black line is generated by correlation analysis.

**Figure 4:**
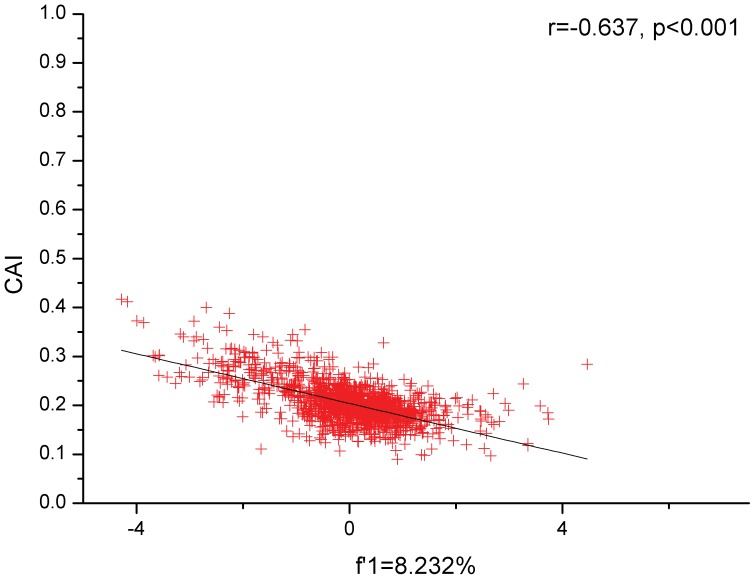
The correlation between CAI values and the first major variation for synonymous codon usage pattern of genes of *M. bovis*. The red plot represents the CAI value against the first major variation for each gene, and the black line is generated by the correlation analysis.

CAI data have a range from 0.417 to 0.09, suggesting that gene expression is generally low. The plot of CAI values vs. GC3s% indicates that genes with considerably low GC3s% possess potential low expression efficiency. Nucleotide compositions, such as GC3s%, play a relatively weak role in influencing gene expression according to the low correlation between CAI and GC3s% (r = −0.196, p<0.001) (Fig. 3). It is interesting that the genes with relatively high CAI values (more than 0.4) have a strong tendency to select some synonymous codons with an A/T-end; however, genes with low CAI values (less than 0.2) tend to select synonymous codons with a G/C-end to some degree. Furthermore, the strongly negative correlation (r = −0.637, p<0.001) between CAI and the first major variation indicates that the overall codon usage pattern of *M. bovis* can sustain its low levels of genes expression (Fig. 4).

### The overall codon usage patterns for some proteins of *M. bovis*

Some proteins related to membrane composition, translational factors, and metabolic factors (membrane proteins, lipoproteins, transposase, 30S ribosomal proteins, and 50S ribosomal proteins) were analyzed according to the overall codon usage patterns (the first major variation and the second major variation) of PCA. The first major variation accounting for the overall codon usage of 30S proteins has a general tendency to be less than zero (Fig. S4), while that accounting for the overall codon usage of 50S proteins does not have this feature (Fig. S5). These results imply that although 30S protein and 50S protein need to coordinate with each other to carry out the corresponding translational function in *M. bovis*, the overall codon usage of 30S proteins in the evolutionary process has a stronger tendency under a certain selection than that of 50S protein. As for the overall codon usage of transposase, the first major variation accounting for the overall codon use of these genes has an obvious tendency to be more than zero (Fig. S6), suggesting that the overall codon usage of these genes points to strong selection. As for the overall codon usage of lipoproteins and membrane proteins, the overall codon usage represents radiation with the origin as the center (Figs. S7 and S8), suggesting that the two types of genes possess a relatively high level of mutations. After analyzing the overall codon usage of the genes with specific functions, these results suggest that even though mutational pressure from nucleotide composition and translation selection drives the synonymous codon usage of genes in the *M. bovis* genome, synonymous codon usage patterns of genes with specific functions represent a specific evolutionary direction.

### The preference of the translation elongation region in genes of the *M. bovis* genome

**Figure 5:**
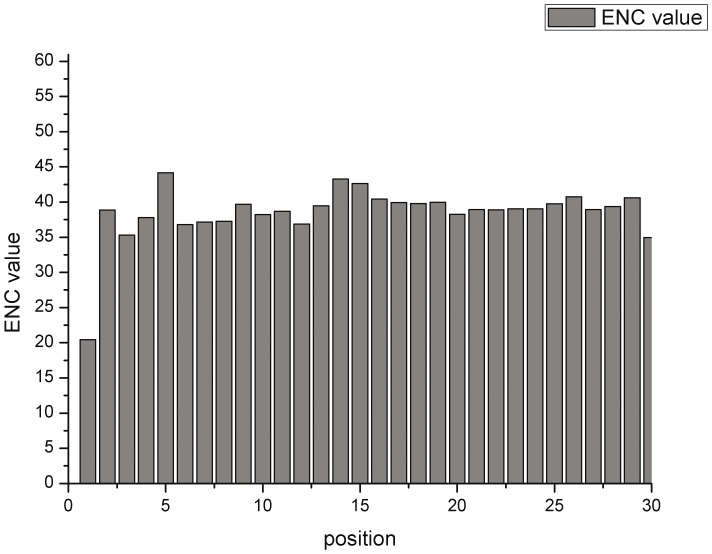
The gradient of overall codon usage bias for each codon position in the translation elongation region of genes of *M. bovis*.

As for the gradient of codon usage preference of the translation elongation region (the first 30 codons of an open reading frame), the ENC data of this beginning part have a range from 20.455 to 44.170 (Fig. 5). The generally low data for the translation initiation region suggest that synonymous codon usage patterns in this region are more stable than ENC values. This indicates that translation selection sustains the relatively conversed synonymous codon usage pattern.

## Discussion

In this study, we analyzed the effect of some evolutionary selections on the synonymous codon usage patterns in *M. bovis*. We found that mutational pressure caused by nucleotide composition, translation selection, and gene expression level shaped the synonymous codon usage patterns in the target organism. However, analyzing the gradient of correlation between different evolutionary factors and the overall codon usage pattern revealed that mutational pressure from nucleotide content dominates over other evolutionary factor. The synonymous codon usage bias responds to the AT- or GC-rich content of the genome, and the synonymous codon usage patterns at least partly account for a mutation-selection equilibrium between the various synonymous codons in specific organisms [30]. Some prokaryotic organisms, such as *M. capricolum*, have an AT-rich (more than 70%) genome and a strong tendency to select CGG for Arg; however, some organisms with a GC-rich genome (more than 70%) tend to select AUA for Ile and AGA for Arg [4], [31], [32]. In *M. bovis*, the codons in which G/C nucleotides occupy two or three positions are generally strictly used in the leading and lagging strands. The significant genetic feature of genes with considerably high A/T in the *M. bovis* genome could be the result of the relative gene expression to carry out translation mechanism because they select an alternate genetic code in which the codon UGA encodes the amino acid tryptophan instead of the usual stop codon and synonymous codons with G/C-ends are strictly selected at a considerably low level [13], [33]. The synonymous codon usage patters of some simple prokaryotic organisms (such as Chlamydiae and spirochaete species) are influenced by the strand-specific mutational bias, which belongs to a type of evolution factor [15], [19]. For the *M. bovis* genome, no evidence suggests the effect of the strand-specific mutational bias on synonymous codon usage for the leading and lagging strands. The G3s% and C3s% have undoubtedly contributed to synonymous codon usage in the leading and lagging strands; however, the G3s% and C3s% weakly influence the synonymous codon usage pattern in the leading and lagging strands, possibly because of the considerably low GC content of *M. bovis* genes. The codon usage, which could respond to the isoacceptor tRNA concentration, reduces the metabolic load and therefore is beneficial to organisms that spend part of their lives under rapid growth conditions [34], [35], [36]. Mycoplasmas are rapidly evolving bacteria as indicated by their position on one of the longest branches of the phylogenetic tree life [37]. This evolution results in drastic genome downsizing, decrease of coding capacity and a limited number of metabolic pathways [38]. After mycoplasmas infecting its natural hosts, these biological characterizations enable mycoplasmas to survive hostile environments and adapt to new niches or hosts [39]. Due to having evolved a series of mechanisms and strategies, *M. bovis* could select the specific pattern of synonymous codon usage by itself. The strong tendency to select optimal synonymous codons assists genes in performing a generally high level of expression in *M. bovis*, and the genetic feature likely provides a clue to the design of heterologous gene expression in some organisms. Previous reports pointed out that the codon usage bias in the beginning part of a coding sequence plays an important role in regulating gene expression [16], [40], [41], [42]. In *M. bovis*, the considerably strong bias of codon usage in the translation elongation region might be sustained under the translation selection and affect gene expression. Apart from the synonymous codon usage bias, the initial open reading frame elongation rate play an important role in the translation level [42], [43].

In conclusion, a series of comprehensive analyses of synonymous codon usage patterns have yielded a basic understanding of mechanisms for codon usage in *M. bovis*. This information could be helpful in future investigations of evolutionary mechanisms in mycoplasma, as well as the cloning and heterologous expression of its functionally important proteins.

## Supporting Information

Figure S1 **The comparison of 59 synonymous codon usage patterns of the leading and the lagging strands of** ***M. bovis*** **.** The X-axes represent the first major variation for each gene of the leading strand, and the Y-axes represent the first major variation for each gene of the lagging strand. The black dots represent the 59 synonymous codon usage patterns. A black dot is located in this red line reflects the same codon usage of a certain synonymous codon of genes on the leading and lagging strands of *M. bovis*.
(TIF)

Figure S2 **The effect of GC3s% of genes on the overall codon usage pattern of** ***M. bovis*** **.** Plots were generated by PCA. The red dots represent genes with GC3s% <10%; the green dots represent genes with GC3s% in the range of 10–20%; the blue dots represent genes with GC3s% in the range of 20–35%.
(TIF)

Figure S3 **The correlation between the ENC value and the first major variation for the synonymous codon usage pattern of genes of** ***M. bovis*** **.** The red dots represent each gene of *M. bovis*. The black line was generated by the correlation analysis.
(TIF)

Figure S4 **The synonymous codon usage pattern of genes for 30S ribosomal proteins of** ***M. bovis*** **.** The blue dots represent genes for 30S ribosomal proteins, and the red dots represent other genes of *M. bovis*. These plots were generated by PCA.
(TIF)

Figure S5 **The synonymous codon usage pattern of genes for 50S ribosomal proteins of** ***M. bovis*** **.** The green dots represent genes for 50S ribosomal proteins, and the black dots represent other genes of *M. bovis*. These plots were generated by PCA.
(TIF)

Figure S6 **The synonymous codon usage pattern of genes for transposases of** ***M. bovis*** **.** The blue dots represent genes for transposases, and the red dots represent other genes of *M. bovis*. These plots were generated by PCA.
(TIF)

Figure S7 **The synonymous codon usage pattern of genes for lipoproteins of** ***M. bovis*** **.** The green dots represent genes for lipoproteins, and the black dots represent other genes of *M. bovis*. These plots were generated by PCA.
(TIF)

Figure S8 **The synonymous codon usage pattern of genes for membrane proteins of** ***M. bovis*** **.** The blue dots represent genes for membrane proteins, and the red dots represent other genes of *M. bovis*. These plots were generated by PCA.
(TIF)

Table S1 **The comparison of the synonymous codon usage pattern between** ***M. bovis*** **and cattle.**
(DOC)
