# Supplementary figures and images for: The Effect of Multiple Evolutionary Selections on Synonymous Codon Usage of Genes in the Mycoplasma bovis Genome
Source: PLoS One. 2014 Oct 28;9(10):e108949. doi: 10.1371/journal.pone.0108949 (PMC4211681; doi:10.1371/journal.pone.0108949)

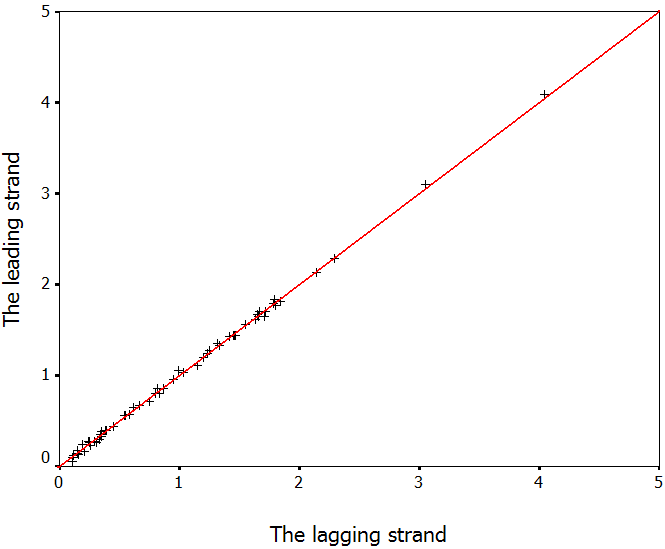

Supplement: Figure S1 — The comparison of 59 synonymous codon usage patterns of the leading and the lagging strands of M. bovis . The X-axes represent the first major variation for each gene of the leading strand, and the Y-axes represent the first major variation for each gene of the lagging strand. The black dots represent the 59 synonymous codon usage patterns. A black dot is located in this red line reflects the same codon usage of a certain synonymous codon of genes on the leading and lagging strands of M. bovis. (TIF) [file pone.0108949.s001.tif]
