# Supplementary material for: The Effect of Multiple Evolutionary Selections on Synonymous Codon Usage of Genes in the Mycoplasma bovis Genome
Source: PLoS One. 2014 Oct 28;9(10):e108949. doi: 10.1371/journal.pone.0108949 (PMC4211681; doi:10.1371/journal.pone.0108949)
Supplement: Table S1 — The comparison of the synonymous codon usage pattern between M. bovis and cattle. (DOC) [file pone.0108949.s009.doc]

**Table S1 The comparison of the synonymous codon usage pattern between *M. bovis*** and cattle

| **Codon (Amino acid)** | **Strain**  **PG45** | **Strain**  **HB0801** | **Strain**  **Hubei-1** | **Host**  **cattle** |
| --- | --- | --- | --- | --- |
| **TTT(F)** | **1.33** | **1.33** | **1.32** | **0.85** |
| **TTC(F)** | **0.67** | **0.67** | **0.68** | **1.15** |
| **TTA(L)** | **3.3** | **3.3** | **3.41** | **0.38** |
| **TTG(L)** | **0.35** | **0.35** | **0.41** | **0.71** |
| **CTT(L)** | **1.53** | **1.53** | **1.41** | **0.70** |
| **CTC(L)** | **0.08** | **0.08** | **0.07** | **1.26** |
| **CTA(L)** | **0.74** | **0.74** | **0.7** | **0.36** |
| **CTG(L)** | **0** | **0** | **0** | **2.59** |
| **ATT(I)** | **2.24** | **2.24** | **2.24** | **0.98** |
| **ATC(I)** | **0.43** | **0.43** | **0.4** | **1.57** |
| **ATA(I)** | **0.33** | **0.33** | **0.35** | **0.45** |
| **GTT(V)** | **2.95** | **2.95** | **2.98** | **0.64** |
| **GTC(V)** | **0.08** | **0.08** | **0.08** | **1.01** |
| **GTA(V)** | **0.76** | **0.76** | **0.75** | **0.40** |
| **GTG(V)** | **0.21** | **0.21** | **0.19** | **1.95** |
| **TCT(S)** | **1.19** | **1.19** | **1.13** | **1.04** |
| **TCC(S)** | **0.81** | **0.81** | **0.87** | **1.37** |
| **TCA(S)** | **0.63** | **0.63** | **0.57** | **0.79** |
| **TCG(S)** | **1.37** | **1.37** | **1.43** | **0.39** |
| **AGT(S)** | **1.97** | **1.97** | **1.96** | **0.87** |
| **AGC(S)** | **0.03** | **0.03** | **0.04** | **1.53** |
| **CCT(P)** | **1.12** | **1.12** | **1.12** | **1.08** |
| **CCC(P)** | **0.88** | **0.88** | **0.88** | **1.39** |
| **CCA(P)** | **1.61** | **1.61** | **1.61** | **1.00** |
| **CCG(P)** | **0.39** | **0.39** | **0.39** | **0.53** |
| **ACT(T)** | **1.23** | **1.23** | **1.19** | **0.89** |
| **ACC(T)** | **0.77** | **0.77** | **0.81** | **1.55** |
| **ACA(T)** | **1.89** | **1.89** | **1.9** | **1.01** |
| **ACG(T)** | **0.11** | **0.11** | **0.1** | **0.56** |
| **GCT(A)** | **1.29** | **1.29** | **1.2** | **1.00** |
| **GCC(A)** | **0.03** | **0.03** | **0.03** | **1.71** |
| **GCA(A)** | **3.07** | **3.07** | **3.28** | **0.80** |
| **GCG(A)** | **0.08** | **0.08** | **0.08** | **0.48** |
| **TAT(Y)** | **0.87** | **0.87** | **0.98** | **0.79** |
| **TAC(Y)** | **0.67** | **0.67** | **0.44** | **1.21** |
| **CAT(H)** | **1.84** | **1.84** | **1.82** | **0.75** |
| **CAC(H)** | **0.05** | **0.05** | **0.03** | **1.25** |
| **CAA(Q)** | **2.11** | **2.11** | **2.15** | **0.46** |
| **CAG(Q)** | **0** | **0** | **0** | **1.54** |
| **AAT(N)** | **1.31** | **1.31** | **1.34** | **0.81** |
| **AAC(N)** | **0.34** | **0.34** | **0.29** | **1.19** |
| **AAA(K)** | **2.33** | **2.33** | **2.37** | **0.78** |
| **AAG(K)** | **0.02** | **0.02** | **0** | **1.22** |
| **GAT(D)** | **2.38** | **2.38** | **2.3** | **0.84** |
| **GAC(D)** | **0.15** | **0.15** | **0.16** | **1.16** |
| **GAA(E)** | **1.4** | **1.4** | **1.45** | **0.78** |
| **GAG(E)** | **0.07** | **0.07** | **0.09** | **1.22** |
| **TGT(C)** | **1.41** | **1.41** | **1.29** | **0.85** |
| **TGC(C)** | **0.59** | **0.59** | **0.71** | **1.15** |
| **CGT(R)** | **3.19** | **3.19** | **2.97** | **0.49** |
| **CGC(R)** | **0.03** | **0.03** | **0.05** | **1.17** |
| **CGA(R)** | **0.02** | **0.02** | **0** | **0.68** |
| **CGG(R)** | **0** | **0** | **0** | **1.32** |
| **AGA(R)** | **2.76** | **2.76** | **2.98** | **1.14** |
| **AGG(R)** | **0** | **0** | **0** | **1.20** |
| **GGT(G)** | **2.34** | **2.34** | **2.44** | **0.64** |
| **GGC(G)** | **0.69** | **0.69** | **0.63** | **1.43** |
| **GGA(G)** | **0.89** | **0.89** | **0.85** | **0.95** |
| **GGG(G)** | **0.08** | **0.08** | **0.08** | **0.99** |
